# Trust, Democracy and Public Health: a fragile pact under pressure

**DOI:** 10.1093/eurpub/ckaf111

**Published:** 2025-07-31

**Authors:** Tit Albreht, Charlotte Marchandise, Hans Henri P Kluge, Margrieta Langins, Sulakshana Nandi, Melanie Hyde, Tomas Zapata, Ilmo Keskimäki, Floris Barnhoorn

**Affiliations:** President of EUPHA; Executive Director of EUPHA; WHO Europe Regional Director; Policy Adviser; Technical Officer; Technical Officer; Chair of the 18th EPH Conference; Deputy-Director EPH Conference

Public health thrives on trust. Trust in science, trust in institutions, trust in each other. It is not a coincidence that the erosion of trust goes hand in hand with the erosion of health: we see it in vaccine hesitancy, in the rise of disinformation, and in the backlash against equity-oriented policies. As public health professionals, we often talk about determinants of health? but rarely do we name democracy as one of the most fundamental.

In recent months, the European public health community has faced an unexpected and deeply concerning challenge: the uncertainty surrounding the future of the EU4Health operating grant. This grant has long been the financial lifeline for many civil society organizations working on health, equity, and prevention. The potential loss or significant reduction of this funding goes far beyond a technical or bureaucratic issue; it threatens the very fabric of our collective European public health ecosystem.

Let’s be clear: this is not only about money. It is about values. It is about whether we, as a Union, continue to believe that strong, independent, science-driven public health voices must be supported and protected—especially when they challenge the status quo, speak truth to power, or defend vulnerable populations.

In a year when European citizens have gone to the polls and new leadership is emerging across EU institutions, we must remind ourselves—and our elected representatives—that democracy is not just about elections. It is about an active, resilient civic space. It is about making room for voices that are not for sale, and for data that are inconvenient. If public health is to remain the backbone of a fair and sustainable Europe, we must not let it be hollowed out by short-term economic logic or political expediency.

We often speak about resilience and how it needs to be enhanced. Resilience needed in view of health challenges, climate change, stress and pressure of everyday life and the rising number of conflicts. And nothing would better improve and enhance individual’s and population’s resilience than investing into better health and well-being at all levels. In that sense, the dižlemma between resilience and health is a non-real one.

The work of public health associations, Non-Governmental Organizations (NGOs), and scientific networks is often invisible—until it disappears. We write guidelines that prevent disease before it emerges. We advocate for healthy environments before crises hit. We bridge the gap between citizens and institutions. And yes, we hold governments accountable when they fail to protect health. If we vanish from the scene, it will be noticed? too late.

This editorial is not a call for pity, but for responsibility. At EUPHA, we are doubling down on what matters: building alliances, investing in youth, strengthening scientific evidence, and championing trust. But we cannot do this alone. We need courageous leadership in Brussels and across Member States to safeguard the role of public health actors in shaping the future of Europe.

In the face of uncertainty, we choose transparency. In the face of cuts, we choose collaboration. In the face of shrinking civic space, we choose voice.

Because health is democracy. And democracy, in turn, must be healthy.

## WHO Europe: Zero tolerance for gender-based violence in the health workforce

KlugeHans Henri P
WHO Europe Regional Director
LanginsMargrieta
Policy Adviser
NandiSulakshana
Technical Officer
HydeMelanie
Technical Officer
ZapataTomas

One in four women and girls in the WHO European Region, experience intimate partner violence or sexual violence in their lifetime [1, 2]. Vital to addressing this violence are health workers who respond to women and their families. And in order to successfully tackle this public health crisis, it is equally important to protect those who provide care [3].

To address the issue of gender based violence (GBV) systematically and at scale, WHO/Europe has launched a Special Initiative to respond to Violence against Women and Girls.

By expanding our focus to include women health workers, many of whom experience violence and discrimination in their workplaces and society, we can tackle head on the need for the health sector to do more to protect them from violence and discrimination at work, while also ensuring that they can access necessary support for experiences of violence outside work.

The need is dire. The scale and volume is such that if addressed, the impact would reverberate across society, given that the European region’s health and care sector is the most feminized workforce of all six WHO regions; 7 out of 10 health and care workers are women [4].

Inequalities persist, nonetheless [5, 6]. Women health workers are concentrated in roles that are undervalued and under-paid and continue to face violence, bullying, micro aggressions, racism, and sexual harassment at their workplace [7, 8].

The statistics are striking. Up to 38% of health workers suffer physical violence at some point in their careers [9]. The 2024 EU gender-based violence survey data shows that one in three women have been sexually harassed at work in their lifetime, with younger women reporting higher prevalence [10]. More efforts are required to understand the risk factors and impacts of sexual harassment and other forms of violence, specifically in the health sector, but regional estimates suggest that among workers in the EU, health professionals are three times more likely to report unwanted sexual attention.

The impact on health systems and societies is debilitating, leading to stress and burnout, impacting mental health, motivation, performance, quality of patient care, and retention.

A number of nursing and medical profession associations, WHO member states and constituents—among them Italy, the Republic of Moldova and Public Health Wales—are revising policies and procedures [11–13]. But at a time when the region faces a health workforce crisis, we must do better [14].

We need to address violence, bullying and systemic discrimination against women in all their diversity and make workplaces safer to retain and attract employees. We need to include prevention and response to violence in education and training, collaborate and engage all stakeholders; from health worker unions to patient bodies and women’s organizations.

Several things need to happen.

To understand the extent, monitor and plan for corrective measures—data gathering on prevalence and impact on the health workforce are required. To address systemic disadvantages—improved working conditions related to fair pay, work-life balance, working time, employment security, and career progression are essential. To enhance workplace safety, establish effective rights-based systems for complaints with a strong institutional response, laws and policies for zero tolerance, guided by international labour standards [15] and the Global health and care workers Compact [16], are a necessity. To enhance gender equality and social cohesion and to diversify leadership—workplaces need Diversity, Equality, and Inclusion initiatives and female leaders of diverse backgrounds.

Change is here already.

All 53 WHO Europe Member states have adopted the central pillar on Retain and Recruit in the Framework for action on the health and care workforce in the Region 2023–30; emphasizing zero tolerance and a need for gender-responsive policies [17].

The European Commission and WHO Europe have launched the Nursing Action with 20 Member States, incorporating dimensions related to violence against health workers. The two actors have also undertaken a first-of-its-kind survey on doctors and nurses’ well-being and mental health across the European Union, Iceland and Norway, to explore the prevalence of 3rd party and physical violence, bullying and sexual harassment.

Violence across the health workforce reflects violence we see across society, disproportionately impacting women and those experiencing intersecting inequalities. But health systems can lead, by transforming themselves to be safer for the workforce and patients.

The WHO Special Initiative is a call to health systems across the region to ensure that prevention and response efforts include a key pillar of health systems: women.

## Welcome to the 18th European Public Health Conference

KeskimäkiIlmo
Chair of the 18th EPH Conference
BarnhoornFloris
Deputy-Director EPH Conference

The 18th European Public Health (EPH) Conference will take place in **Helsinki, Finland, from 11–14** November 2025. Pre-conferences are scheduled for Tuesday, 11 November, followed by the main conference from 12 until 14 November. The theme for 2025 is: **‘Investing for Sustainable Health and Well-Being’.**

### Investing for Sustainable Health and Well-being

By promoting integrated and sustainable health approaches, the conference aims to inspire policies and practices that support health equity, environmental sustainability, and social justice. Investing in health and well-being is crucial for building resilient communities and a sustainable future. Health should be seen as an investment, not an expense.

In recent years, Europe has faced significant challenges, with many governments losing sight of crucial issues for their countries and the world. In too many parts of the world, war and violence have re-emerged, causing immense suffering and becoming major public health threats. While peace efforts are self-evidently the most urgent public health tasks, other critical public health issues have also been neglected far too long. Overwhelming evidence shows that ecological and social sustainability is essential for the future of our planet and humanity. However, despite knowing what needs to be done to achieve this, changing outdated economic, social, and health policies remains difficult. The upcoming EPH Conference emphasizes this need for societal change.

In addition to the extensive plenary programme, the conference offers 16 Conference Tracks covering varying fields of public health. The track closest to the theme of the conference is Track A ‘The Economy of Well-being and Public Health’. This track contains nine workshops of which we want to highlight three here.

**Well-being economy and public health: investing in prevention for prosperity, resilience, and equity**, organized by the WHO Collaborating Centre on Investment for Health and Well-being at Public Health Wales, UK, provides examples and insights into good practices of policies and investments that improve population health and well-being, enable early prevention, and reduce health inequalities. It includes deep dives into the approaches taken on national and sub-national levels in Finland, Iceland, Wales, Scotland (UK), and Utrecht Region (The Netherlands). These countries/regions are recognized by the WHO as champions of employing well-being economies to promote and improve health, sustainability, and equity outcomes in Europe, providing inspiration for others and collaborating to advance this agenda forward.

**Public health economics at the heart of well-being economics**, organized by EUPHA Public health economics section, focuses on the transition from an economy centred on economic growth to one centred on well-being makes public health central to political and economic priorities. However, public health economics is rarely used as a concept and as a central part of welfare economics debates. This round table will critically reflect on the role of public health economics as a transformer of public policy and economic evaluation. The main objective is to understand and discuss how current public health economics tools can be adapted and extended to guarantee public policies that promote well-being throughout the life cycle, but also equity and sustainability.

**Reimagining how to plan investment in health promotion and disease prevention**, organized by Invest4Health, an EU Horizon Europe funded project. Prevention and investment are not terms that have often been combined. Indeed, securing sufficient investment for health promotion (and disease prevention) is a perpetual challenge. In a working environment with increasing scarcity of resources and static public budgets, innovative approaches are needed to maximize public sector assets and perhaps blend this with private investment options including from health insurers. The reason for this workshop is that in an economy of well-being, investing in prevention rather than repair offers a more sustainable return on investment for the economy and its communities. Paradoxically, the 2007–09 economic/financial crises and the COVID pandemic also reminded us of two lessons learned by government and municipalities since the 19th century; the centrality of health in our societies and economies and the importance of prevention as a strategic principle for planning and investment. Prevention is an investment and needs to be designed and financed within an investment paradigm. Accordingly, the workshop will share learning and insights from the EU Horizon project ‘Invest4Health’. The project has been developing and testing finance and investment models, an alternative governance mechanism and practical tools, that will start to show how this shift can be accomplished using ‘smart capacitating investment’.

You’re welcome to attend these workshops and join the discussion.

We look forward to welcoming you to Helsinki!
